# Silencing Sexually Transmitted Infections: Topical siRNA-Based Interventions for the Prevention of HIV and HSV

**DOI:** 10.1155/2014/125087

**Published:** 2014-01-12

**Authors:** Lee Adam Wheeler

**Affiliations:** M.D./Ph.D. Program, Harvard Medical School, Program for Cellular & Molecular Medicine, Boston Children's Hospital, Boston, MA 02111, USA

## Abstract

The global impact of sexually transmitted infections (STIs) is significant. The sexual transmission of viruses such as herpes simplex virus type-2 (HSV-2) and the human immunodeficiency virus type-1 (HIV-1), has been especially difficult to control. To date, no effective vaccines have been developed to prevent the transmission of these STIs. Although antiretroviral drugs have been remarkably successful in treating the symptoms associated with these viral infections, the feasibility of their widespread use for prevention purposes may be more limited. Microbicides might provide an attractive alternative option to reduce their spread. In particular, topically applied small inhibitory RNAs (siRNAs) have been shown to not only block transmission of viral STIs to mucosal tissues both *in vitro* and *in vivo*, but also confer durable knockdown of target gene expression, thereby circumventing the need to apply a microbicide around the time of sexual encounter, when compliance is mostly difficult. Despite numerous clinical trials currently testing the efficacy of siRNA-based therapeutics, they have yet to be approved for use in the treatment of viral STIs. While several obstacles to their successful implementation in the clinic still exist, promising preclinical studies suggest that siRNAs are a viable modality for the future prevention and treatment of HSV and HIV.

## 1. Clinical Context

Sexually transmitted infections (STIs) continue to be a major source of global morbidity and mortality [1]. Viral infections, notably herpes simplex virus type-2 (HSV-2) and the human immunodeficiency virus type-1 (HIV-1), have proven particularly problematic to control from both scientific and public health perspectives.

HSV-2, the most common cause of genital ulcers, is the most widespread viral sexually transmitted infection (STI) worldwide [2]. Prevalence is high; currently estimated to be ~15–25% of sexually active adults in the US and as high as 60–80% in some developing nations [2–5]. Rates of infection are even higher among women, owing to the greater efficiency of male-to-female (MTF) transmission [6, 7]. While multiple variables factor into the high prevalence rate at the population level, the fact that most sexual transmissions occur in the absence of clinically identifiable genital lesions likely plays a major role [8, 9]. While antiviral drugs have proven effective to reduce viral transmission [6, 8, 10], they are not curative. Furthermore, due to the high costs associated with population-wide screening and maintenance therapy, their utility as public health initiatives to reduce transmission is debatable [11]. This is especially true when more economical interventions, such as condoms, are known to be similarly effective for prevention purposes [12, 13]. Nonetheless, the numbers of infected individuals continues to rise underscoring the need for developing new modalities to reduce transmission and clear what are currently life-long infections.

Epidemiological analysis has established a link between the prevalence of HSV-2 and HIV-1 [3]. In fact, individuals infected with HSV are at greater risk of acquiring HIV after exposure, underscoring the fact that herpes infection is an important cofactor for HIV transmission [4, 14–16]. While the prevalence of HIV-1 is much lower than that of HSV-2, the global burden of HIV is significant. An estimated 60 million people have been infected by the virus, and some 30 million people are currently living with HIV [17]. Research on HIV vaccine development began almost immediately after HIV was identified as the causative agent for AIDS [18–21]. However, to date, no effective vaccines against HIV have been developed [22–24]. Since 1996, there has been a dramatic decline in the clinical burden associated with HIV infection with the introduction of highly active antiretroviral therapy (HAART), an approach predicated on the simultaneous pharmacological inhibition of multiple components of the viral life cycle [25, 26]. HAART has significantly reduced both the mortality and morbidity associated with HIV and it is now the standard of care for the treatment of HIV in both children and adults [27–30]. Continued challenges to HIV control include drug resistance, poor compliance, and limited access, especially in the developing world [31–43]. Importantly, due to the persistence of viral reservoirs in infected individuals, HAART cannot eliminate virus from the seroconverted host [44–48].

Therefore, a combination of therapeutic and preventative approaches is needed to effectively manage the growing epidemic [49]. While preexposure prophylaxis (PrEP) has proven an effective approach to preventing transmission [50, 51], high cost, potential side effects, and possible development of drug resistance suggest that PrEP may be best suited to targeted use in only the highest-risk populations [52, 53]. That being said, given that 2.7 million people were newly infected with HIV in 2010 alone [54], the continued spread of the infection underscores the urgent need for the development of novel ways to prevent viral transmission.

An extensive body of knowledge and research that has accumulated over the past decades on viral transmission and pathogenesis, however HSV-2 and HIV-1 continue to present formidable scientific and public health challenges. Vaccines are often considered the gold standard for prevention of viral infection; but historically, vaccine strategies against viral STIs have proven ineffective [55–61]. While more recent vaccine candidates against HIV-1 and HSV-2 have shown a modest protective effect, none have yet been FDA approved for use. In fact, the vaccine against the human papilloma virus (HPV) is the only vaccine currently available and approved for the prevention of a viral STI [62–65]. While its cost is still prohibitive in most developing countries, it has proven highly effective against the primary strains of HPV known to cause both genital warts and cervical cancer. In the absence of an effective vaccine against HSV-2 and HIV-1, the global burden of disease, cost of treatment, and lack of cure all serve to highlight the need for research into alternative interventions to prevent the transmission of viral STIs.

## 2. STI Microbicides

One particularly attractive option is a topical microbicide, which could reduce sexual transmission of the virus at the mucosal site of entry [66, 67]. Several microbicidal compounds targeting HIV-1, HSV-2, and HPV are being assessed in clinical trials, and more are currently under investigation [68].

The earliest microbicide candidates were surfactants, such as nonoxynol-9 (N9), that showed antiretroviral activity against both HIV and HSV *in vitro *by disrupting the membrane coat of the virus [69–71]. When applied intravaginally, however, N9 led to higher rates of HIV infection [72–75]. Because this was most likely secondary to the inflammation it induces in the genital tract, further studies against both HIV and HSV were subsequently abandoned.

Over the past two decades of research in the field, multiple alternative vaginal microbicide strategies—ranging from acid buffering gels [76] to polyanionic inhibitors of virus entry [77]—have been tested both *in vitro* [78] and clinically [79] with little success. Most rectal formulations have been based on those models successful in preclinical trials for vaginal application and have met with a similar fate. To date, only one has demonstrated clinical efficacy, both against HIV and HSV-2 [80]: a vaginally applied 1% tenofovir gel, which was the first microbicide tested clinically that afforded study participants protection from viral transmission. While protection was only partial, CAPRISA004 served as the catalyst for further study on topical tenofovir gels, including those intended for rectal administration [81, 82]. While follow-up studies have failed to reproduce the partial protection observed in CAPRISA004 [83], post hoc data analysis suggests that this was due in large part to inadequate compliance of study subjects to the treatment regimen. The future of tenofovir gel as a preventative microbicide remains unclear, but this first success story in the decade-long history of research in microbicide development has galvanized interest in working toward designing a viable topical microbicide to prevent the sexual transmission of HSV and HIV.

## 3. The RNAi Revolution

RNA interference (RNAi) is a cost-effective means of suppressing the expression of virtually any gene in a sequence specific manner [84, 85]. The field began early in the 1990s, with the discovery that small RNA molecules had the ability to regulate developmental timing in the nematode *Caenorhabditis elegans *and trans gene expression in plants [86–88]. Nonetheless, it was the seminal discovery by Fire and colleagues in 1998 that conclusively demonstrated how these RNAs could be used to modulate gene expression [85]. The term RNA interference, or RNAi, came to refer to the process of introducing small dsRNA, termed small inhibitory RNAs (siRNAs), to silence gene expression in a sequence specific manner. When it was later demonstrated to function similarly in mammalian cells, we came to understand it as well-conserved endogenous gene regulatory mechanism that could be harnessed as an experimental tool for the analysis of gene function. In so doing, it also opened the door for the development of novel therapeutic approaches for multiple diseases, including viral pathogens (e.g., HIV-1 [89–93], respiratory syncytial virus [RSV] [94], and herpes simplex virus-2 [95, 96]), inflammatory disorders [97–100], and a variety of cancers [101, 102].

**Figure 1:**
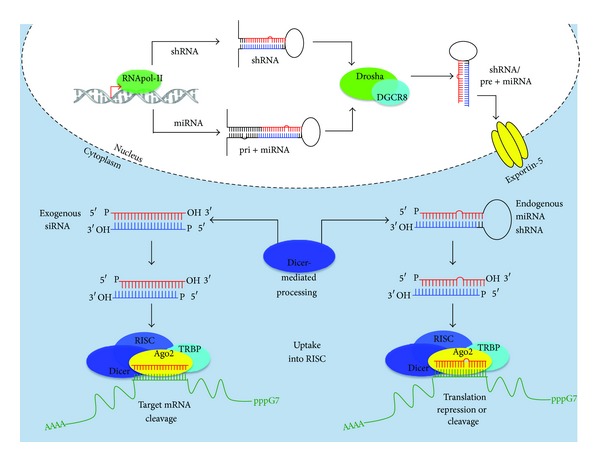
Cellular machinery for RNAi-mediated gene silencing. (a) miRNA-mediated gene silencing. miRNAs (endogenous) and shRNAs (exogenous—lentiviral vectors) are expressed from Pol II-derived primary transcripts (pri-miRNA/pri-shRNA). The pri-miRNA/shRNA is cleaved by Drosha, an RNase III-type enzyme, producing the precursor miRNA/shRNA (pre-miRNA/shRNA), which is exported into the cytoplasm by exportin-5. Once in the cytoplasm, the loop of the pre-miRNA is cleaved off by Dicer, producing the transiently double-stranded miRNA, which is taken up by the miRNA-containing RISC (miRISC). The passenger strand rapidly dissociates, leaving the single-stranded mature miRNA to guide the sequence-specific inhibition of translation or cleavage of the target mRNA. shRNA is processed in a similar way but has been introduced to the host genome exogenously, often by lentiviral vector. (b) siRNAs, 19–21 nucleotide duplexes, are processed by Dicer to generate 2-3 nucleotide overhangs at their 3′ ends and phosphate groups on their 5′ termini., thereby activating the siRNAs. Active siRNAs are then incorporated into the RISC. The passenger strand of the duplexed siRNA is cleaved and released from the complex, leaving the single-stranded guide strand to direct RISC to the complimentary site on the target mRNA. Ago2 catalyzes the cleavage of target mRNA in trans, and the cleaved mRNA is released and the active strand containing RISC can direct the cleavage of additional target mRNAs. Ago: argonaute; RISC: RNA-induced silencing complex. Figure is adapted from [105].

Therapeutically relevant siRNAs can be introduced to the endogenous RNAi pathway at various stages (Figure 1). For example, siRNAs can be derived from short hairpin sequences (shRNAs) introduced to the host genome by viral vectors [103], which follow much the same processing pathways as endogenous microRNAs (miRNAs). After transcription, shRNAs are cleaved in the nucleus by the RNAse III Drosha, after which time they are exported into the cytoplasm via exportin-5, and processed by Dicer into siRNAs [104]. Synthetic siRNAs, introduced exogenously to the cytoplasm, bypass much of the host RNAi machinery, converging at the point of Dicer-mediated processing [84].

Both siRNAs and miRNAs that are produced by Dicer act as effector molecules that, through their association with the RNA-induced silencing complex (RISC), guide the sequence-specific inhibition of gene expression. The critical component of RISC is an argonaute (Ago) protein that not only provides the siRNA/miRNA binding site, but also contains the endonucleolytic domain that cleaves complementary mRNA targets.

While, structurally, the siRNA/miRNA strands are symmetric, functionally, they are not equivalent [106]. The strand exhibiting the lower thermodynamic stability upon biding to RISC predominates as the active (antisense) strand used to guide the silencing of target mRNA expression, while the inactive (sense) strand is either cleaved or released from the complex [107]. Strand bias is a critical feature in siRNA design, since it determines target specificity. Most perfectly complimentary sequences (like most siRNAs) allow for site-specific cleavage of the target mRNAs by argonaute 2, an RNAse III enzyme, at predictable and verifiable locations, namely, distal to the 10th nucleotide from the 5′-end of the guide strand [104, 108]. Cleaved target mRNA is subsequently released from the complex and degraded, allowing for the activated RISC to effect subsequent rounds of cleavage. Originally, imperfectly complimentary sequences (like some miRNAs) were thought to only block downstream translation of mRNA strands [109]. Recent evidence suggests that some target cleavage does indeed occur [110, 111], however more robust target gene silencing is observed with perfectly homologous sequences and, thus, will be the focus of this review.

## 4. Antiviral Activity of RNAi

**Figure 2:**
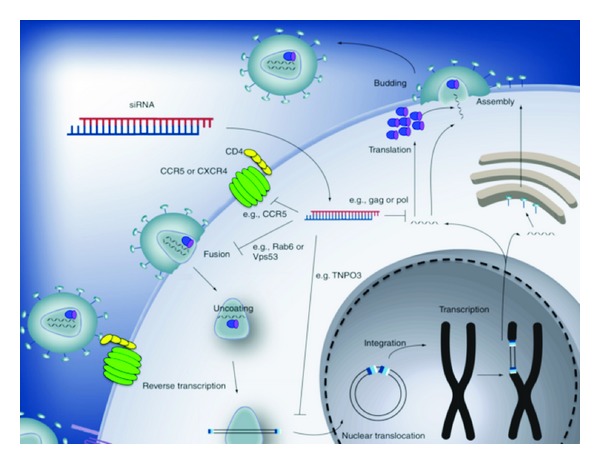
siRNAs directed against either conserved HIV-1 sequences or essential host factors (EHFs) could impair HIV-1 replication at various points in the viral life cycle.Although the incoming viral RNA is protected from siRNA-mediated degradation by its association with the preintegration complex (PIC) [119], it is possible to interfere with EHFs required for viral entry or integration by targeting its receptor CD4, its coreceptor, or factors required for entry into the nucleus such as transportin-3. Targeting conserved HIV-1 sequences can also inhibit progeny virus formation by cleaving HIV-1 transcripts such as gag and pol, expressed by the integrated proviral DNA. Figure is adapted from [105].

Soon after RNAi was shown to operate in mammalian cells, several groups demonstrated that small inhibitory RNAs (siRNAs) could be used to interfere with both of the transmission and replication of HIV-1, HSV-2, and HPV *in vitro* and *in vivo *[112, 113]. For HIV infection, siRNAs can inhibit viral replication *in vitro* by either directly targeting sequences of viral gene products [105, 114–117] or silencing essential host factors (EHFs) [118], such as the HIV receptor CD4 [89] or the CCR5 coreceptor [92] required for productive infection (Figure 2). For antiviral siRNAs, targeting a single conserved HIV-1 sequence is able to inhibit primary isolates from all viral clades [90]. Accordingly, a judicious selection of siRNAs directed against highly conserved viral gene products might allow for the simultaneous inhibition of diverse strains of HIV present throughout the world. Similar approaches have been validated for HSV-2 infection, where siRNAs directed against the nectin-1 cell surface receptor required for viral entry, and viral gene products UL27/UL29 essential to the viral life cycle, effectively inhibit replication. Importantly, the inhibitory effect is more pronounced when targeting both host and pathogen gene products, suggesting that synergistic effects could play an important role.

Early approaches focused primarily on systemic delivery. While this delivery method has potential utility for postinfection therapeutics, systemic administration is unlikely to prevent infection at the site of entry. The proof of principle that topically-applied siRNAs are taken up and effect gene silencing at the mucosa was first shown using a model of respiratory syncytial virus (RSV) infection [94, 120, 121], confirming that siRNA-mediated protection from viral infection is in fact possible in mucosal tissues.

Establishing stable viral resistance in the genital tract to prevent sexual transmission of viruses may be possible based on recent studies of other sexually transmitted infections (STIs). Using a mouse model of herpes infection, intravaginal (IVAG) application of siRNAs targeting HSV-2-encoded genes and/or its cellular receptor, nectin-1, inhibited sexual transmission of HSV-2 for more than one week [95, 96]. Similarly, siRNAs against the CCR5 coreceptor and viral gene products gag and vif have demonstrated protection from vaginal infection in a newly developed small animal model for HIV infection [122, 123].

To address the considerable problem of patient compliance faced by most microbicidal intervention strategies, ensuring durable gene silencing is critical, since lasting knockdown would likely circumvent the need to apply the microbicide prior to each sexual encounter [124]. Two approaches to date have proven effective in this regard. Conjugation of siRNAs to cholesterol moieties prolonged half-life in the vaginal mucosa of mice challenged with HSV-2, maintaining protection even when viral challenge is delayed [96]. For HIV, similar approaches were not possible since the immune cells HIV infects are refractory to transfection with cholesterol conjugated siRNAs. While siRNAs directed against CCR5 were known to effectively silence target gene expression *in vitro* and prevent viral replication in primary macrophages when challenge is delayed for up to 3 weeks [89], only recently was testing possible in an *in vivo *model. Using CD4 aptamers as targeting vehicles, siRNAs against CCR5 were shown to protect from vaginal transmission of the virus when applied 4-5 days prior to viral challenge, and inhibition of viral replication was observed when applied up to one week prior to challenge [122]. While additional testing will be required to confirm these preliminary studies, these data suggest that repeated siRNA applications may have an additive effect. In other words, they may confer durable protection from STIs such as HIV when applied every few days, thereby obviating the need for application just prior to sexual encounters, when compliance is most difficult.

## 5. RNAi Therapeutics: The Challenge of Delivery

Despite the potential promise of antiviral siRNAs for therapy or prevention, the primary obstacle to their widespread clinical application has been delivery; more specifically, finding an efficient means of shuttling exogenous siRNAs across the plasma membrane [112]. Over the years, several techniques have been employed to successfully deliver siRNAs to cells for gene knockdown, including cationic lipids, lentiviral vectors, and conjugation to other molecules such as PEG or cholesterol [125–132]. While these approaches successfully overcome the first barrier to effective siRNA delivery, that is, the plasma membrane, they typically fail to target siRNAs to the cells of interest; that is, they lack cell specificity.

Targeted siRNA delivery may not be essential for all potential clinical applications. Nonetheless, targeting siRNA therapeutics to a specific subset of cells or tissue types may have some significant advantages relative to non-specific administration. In particular, it would minimize non-specific effects and toxicity in bystander cells; and, perhaps more importantly, it would reduce the dose required for therapeutic silencing in the cells and tissues of relevance. For viral STIs, targeted delivery has not been necessary for inhibiting HSV-2. When applied topically to the genital mucosa, the epithelial cells HSV-2 infects readily endocytose both naked and cholesterol conjugated siRNAs, which effect target gene silencing after uptake. HIV has been proven more difficult to prevent in a similar way, primarily because the CD4+ immune cells HIV infects are particularly refractory to most traditional methods of transfection [89, 92]. As a result, siRNA-based interventions have had to overcome the obstacle of *in vivo *transduction of CD4+ cells.

Targeted delivery has become possible in recent years using bifunctional fusion proteins, which couple a targeting antibody fragment (Ab) to an RNA binding motif, such as protamine. Using Fabs recognizing cell surface receptors, such as the lymphocyte function-associated antigen (LFA-1) [98] or the HIV glycoprotein gp120 [93], functional siRNAs have been effectively delivered to relevant cell types, even *in vivo* [91]. Despite these promising results, critics of the antibody-based approach cite difficulties with synthesis, high manufacturing and storage costs, chemical instability, and potential immunogenicity as important limitations for widespread use. Given that HIV-1 and HSV-2 are most prevalent in the developing world and given the economic and storage requirements, they may not be ideally suited for use as preventative microbicides, especially in resource poor settings.

More recently, small structured oligonucleotides, called aptamers, provide a promising alternative option for siRNA delivery. Aptamers can be efficiently and economically synthesized in large scale, and they are amenable to multiple chemical modifications. Moreover, because they are rarely immunogenic, aptamers are ideally suited for therapeutic applications like topical microbicides, which may require repeated administration. Synthesized from randomized oligonucleotide libraries, aptamers are *in vitro* selected for their ability to bind a desired ligand with high affinity. They have been used in several disease models [133], including HIV, primarily for their receptor-blocking properties [134, 135].

Aptamers were recently demonstrated as potential delivery vehicles for siRNA. Using a ligand-targeting aptamer fused at its 3′-end to an siRNA duplex, these aptamer-siRNA chimeras (AsiCs) were first used to transfect and silence gene expression in prostate cancer cells both *in vitro* [136] and *in vivo* [137] using a prostate-membrane specific antigen (PSMA) aptamer. AsiCs, constructed using a gp120-targeting aptamer, were tested *in vitro *and, more recently, *in vivo* [138–140] and reduced viral replication in infected hosts. A second strategy using CD4-targeting aptamers has also demonstrated effectiveness by both inhibiting HIV replication and blocking transmission of the virus *in vivo* [122, 123]. Merging both approaches would likely be even more effective, by coupling a method to prevent *de novo *infection in CD4+ cells susceptible to HIV infection with another that effectively inhibits viral replication in the small number of cells that may become infected with HIV, the virus after exposure. Combinatorial approaches, known to be highly effective against viruses such as HIV given past experience with HAART, capitalize on the aptamer's ability to block HIV entry at CD4, as well as specifically to deliver siRNAs that inhibit factors required for both viral transmission and replication.

## 6. Future Prospects

From its first discovery, less than 20 years ago, the field of RNA interference has evolved at a rapid pace. The potential to silence the expression of virtually any gene product has found applications outside of the lab and has now been successfully applied to the treatment and prevention of several diseases, including viral STIs. Despite these successes, a number of challenges remain for the effective application of RNAi-based approaches in the fight against HIV and HSV. The brisk development of technological innovations in the field has in many ways outpaced our understanding of basic biology of RNAi. As we better understand the underlying biochemical pathways, we can improve the design, the effectiveness, and the side effect profile of these reagents to maximize their therapeutic index. Furthermore, for both therapeutic and preventative purposes, multiple applications over long periods of time may be required. While largely dependent on patient-specific factors such as burden of disease and risk of exposure, this underscores the need for further studies investigating the effects of repetitive administration and long-term use.

The most well-developed therapeutic approaches using siRNA have used localized delivery to easily accessible organs and tissues, including treatment of the eye for age-related macular degeneration or the treatment of lungs for RSV infection [105]. While HSV-2 appears to be effectively controlled with similar tissue-based applications, HIV-1 poses a more difficult challenge. Because it infects multiple cell types that are spread throughout the body, utilizing new modalities (antibodies, aptamers, etc.) to deliver functionally active siRNAs to specific CD4+ target cells will likely be required for future siRNA-based approaches to be effective. Irrespective of the specific delivery mechanism used, the combination of multiple siRNAs targeting either multiple conserved sites on the HIV-1 genome or viral and host factors will undoubtedly minimize the potential for viral escape mutations and, thus, be the most effective strategy. While targeting new host factors implicated in disease pathogenesis could prove useful to identifying new ways to prevent viral transmission, care must be taken in selecting such targets to minimize off-target effects.

While the mouse model has been indispensible for studying topical siRNA-based interventions for both HSV-2 [95, 96] and HIV-1 [122, 123, 141, 142], translating these results into safe and cost-effective therapies for clinical use in humans remains a significant challenge. Nonhuman primate (NHP) models have traditionally been used to bridge the gap between small animal models and clinical trials, especially in STI research; however, meaningful differences between NHP and patient populations do exist. In particular, off-target effects can manifest themselves in vastly different ways from one species to the next and can, during preliminary clinical trials, derail what had been a very promising drug candidate in preclinical studies. Safety concerns are not limited to animal models, however, as the recent termination of the phase III trial targeting macular degeneration using VEGF-specific siRNAs has highlighted [143]. Along with other studies, this has underscored potential bias introduced by nonspecific effects and reinforced the need to rigorously demonstrate siRNA-mediated mechanisms of action at every stage of development. In the case of RNAi-based therapeutics, this becomes especially important when specific chemical modifications have been made or new moieties have been conjugated to siRNA strands for targeting, stability, or other purposes [131, 144, 145].

Given that RNAi-based therapeutics are predicated on the introduction of exogenous RNA to the cytoplasm, it is perhaps not surprising that some reports have found that siRNAs can trigger a type I interferon (IFN) response. Triggering toll-like receptors that recognize intracellular nucleic acids activates a nonspecific immune response that generally blunts knockdown of target genes and produces unwanted side effects [146–148]. While, in certain circumstances, a nonspecific IFN response can enhance the observed therapeutic effect [149, 150], generally, strategies focus on minimizing these off-target effects. To date, substitutions such as 2′-OMe linkages [151], modified DNA/RNA bases [152, 153], and design enhancements to improve the chemical stability of the siRNA moiety [154] have been employed with some success. Nonetheless, reports exist in which modifications of this type did not blunt the innate immune response [155], suggesting that alternative approaches to “mask” siRNAs from TLR recognition would be required [131, 156].

Overexpression of small RNAs can also be problematic. Typically associated with lentiviral shRNA constructs, overexpression can result in saturation of the endogenous RNAi machinery, perturbations of normal miRNA function [157], and even fatal brain toxicity in small animal models [158]. While using siRNAs instead of shRNAs can circumvent these adverse outcomes [159], high concentrations of siRNAs have similar detrimental effects [157]. This underscores the need to improve the potency of siRNA-based constructs while minimizing potential off-target effects. While biochemical and structural modifications have been used to improve potency with some success [151, 152, 160, 161], combinatorial approaches to target gene silencing have also proven useful to reduce the effective concentration of siRNA required for therapeutic effect. Combinatorial approaches do increase competition for RISC loading [94, 162] and thus may result in impaired silencing of one or more target genes depending on factors such as relative chemical stability and affinity for the RISC.

Given the increasing incidence of worldwide infections with both HIV-1 and HSV-2, developing new ways to prevent transmission is essential. Topically applied siRNAs have been shown to not only block transmission of the virus to tissue both *in vitro* and *in vivo* but also confer durable knockdown of target gene expression [95, 96, 122, 123, 163]. Patient compliance will determine the success of any therapeutic intervention, including a topical microbicide, as recent clinical trials testing the efficacy of tenofovir-based gels have demonstrated [80, 83]. These findings underscore the importance of developing interventions and dosing schedules that are not too burdensome to the user, in order to facilitate adherence. Agents that confer durable protection might circumvent the need to apply a microbicide around the time of sexual encounter, when compliance is most difficult [79, 124]. In some cases then, RNAi—which has already been shown to knock down multiple target genes in various disease models for several weeks at a time both *in vitro *and *in vivo* [92, 95, 96, 99, 105]—may be an ideal choice. Despite numerous clinical trials currently underway to evaluate the efficacy of RNAi-based therapeutics [164], they have yet to be approved for use in the treatment of viral STIs. While several obstacles to their successful implementation in the clinic still exist, reassuring data highlights the future promise of RNAi as a viable modality for the prevention and treatment of HIV and HSV.

## Conflict of Interests

The author declares no conflict of interests.
